# An invasive *Aspergillus flavus* infection in an extremely preterm neonate: a case report

**DOI:** 10.1016/j.mmcr.2025.100757

**Published:** 2025-12-11

**Authors:** Neema Pithia, Margie Morgan, Thea Tagliaferro, Priya R. Soni

**Affiliations:** a Division of Pediatric Infectious Diseases, Department of Pediatrics, University of California, Los Angeles, Los Angeles, CA, 90095, USA; b Division of Microbiology, Department of Pathology and Laboratory Medicine, Cedars-Sinai Medical Center, Los Angeles, CA, 90048, USA; c Division of Neonatology, Department of Pediatrics, Cedars-Sinai Guerin Children's, Los Angeles, CA, 90048, USA; d Division of Pediatric Infectious Diseases, Department of Pediatrics, Cedars-Sinai Guerin Children's, Los Angeles, CA, 90048, USA

**Keywords:** Neonatal fungal infection, *Aspergillus flavus*, Disseminated fungal infection, Nosocomial fungal infection, NICU infection control, Extremely preterm infant, Angioinvasive fungi, Neonatal sepsis

## Abstract

Extremely preterm neonates are at heightened risk for invasive fungal infections. We report a fatal case of *Aspergillus flavus* infection in a 25-week infant, outlining the clinical course, antifungal treatment, and challenges in management. This case highlights the need for early recognition, rapid biopsy and aggressive therapy in vulnerable neonates.

## 1 Introduction

Invasive fungal infections (IFIs) in neonates are rare but often devastating. *Candida* species remain the most common pathogens; however, cases of neonatal aspergillosis have been reported [1]. *Aspergillus* species are ubiquitous molds present in soil, plants, air and food [1]. *Aspergillus fumigatus* is the predominant cause of neonatal disease, with *A. flavus* and *A. niger* reported less frequently*.* Risk factors for IFIs include extreme prematurity, birth weight ≤1000g, prolonged broad-spectrum antibiotics, and infusion of lipid emulsions [1]. Unique to *Aspergillus* species, outbreaks have been linked to hospital construction and spore carriage on synthetic materials [2]. Experimental models suggest *A. flavus* may be more virulent than *A. fumigatus* [3].

## 2 Case presentation

The patient was a diamniotic monochorionic twin born via emergent caesarean section at 25 weeks and 2 days gestation for maternal pre-eclampsia with severe features. The amniotic fluid was clear with no prolonged rupture of membranes. Maternal prenatal labs were unremarkable. At birth, the infant was intubated in the delivery room and quickly managed with high-frequency oscillatory ventilation. She received two doses of surfactant and parenteral nutrition with lipids via a central line. Empiric ampicillin and gentamicin were administered for 36 hours.

**Fig. 1:**
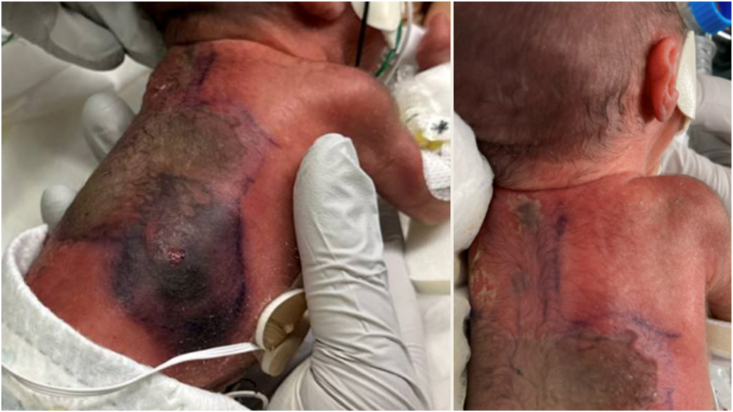
Rapidly progressive violaceous and necrotic lesions on the infant's back with central darkening concerning for invasive fungal infection.

On day of life six, a small ecchymosis appeared on the right flank, progressing rapidly over 6–12 hours to involve the entire back. We will define this as Day 0 (D0) of disease presentation (Fig. 1). She was emergently transferred to a level IV NICU for access to surgical services due to concern for possible invasive infection. A wound culture obtained prior to transfer showed no organisms on gram stain and no fungal growth on culture. Labs at that time revealed WBC 21.1 x 10^3/μL, platelets 146 x 10^3/μL and CRP 4.5 mg/L. She was started on broad spectrum antibiotics as well as liposomal amphotericin B 10mg/kg daily after transfer. Frozen section biopsy performed promptly after transfer demonstrated fungal elements concerning for *Rhizopus* and *Aspergillus.* Extensive and prompt surgical debridement of the back was performed within 6 hours of transfer, with four additional debridements on day one and two of disease presentation (D1 and D2). Antibiotics were discontinued on D2.

**Fig. 2:**
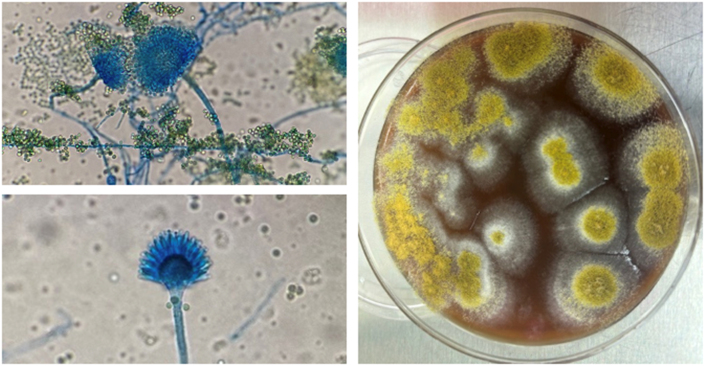
a) *A. flavus* produces hyaline septate hyphae with vesicles that support phialides with spore production and b) *A. flavus* grows well on routine fungal media producing a powdery yellow-green colony within 7 days at 30 °C.

Despite aggressive management, her status worsened with escalating sedation, pain control, and vasoactive support. Micafungin 8mg/kg daily was added on D4 due to clinical deterioration. On D6, fungal cultures demonstrated growth of *Aspergillus flavus* and definitive species identification was subsequently confirmed via MALDI-TOF, prompting a switch to Voriconazole 6mg/kg q12h (Fig. 2). Head ultrasound (HUS) on D6 showed a large right-sided anterior cerebral artery (ACA) infarction; by D8 her HUS showed progressive cerebral infarction (ACA then middle cerebral artery (MCA) territory with hemorrhagic transformation and midline shift). These findings were concerning for intracranial angioinvasive disease. Despite maximal medical and surgical care, she transitioned to comfort care and passed on D10. In vitro susceptibility testing was performed at University of Texas Health San Antonio and returned after the infant's demise (Table 1).

**Table 1:** Minimum inhibitory concentration (MIC) for anti-fungal agents against *A. flavus* isolate.

Anti-fungal Agent	MIC
Amphotericin B	4 μg/mL
Micafungin	≤0.015 μg/mL
Voriconazole	0.5 μg/mL
Posaconazole	0.125 μg/mL
Itraconazole	0.06 μg/mL
Isavuconazole	0.5 μg/mL

The mother's placenta was free of fungal elements; additionally, the patient's twin, who had passed two days prior to D0 from pulmonary hemorrhage, had no evidence of fungal disease at autopsy. In contrast, our patient's autopsy demonstrated residual fungal involvement confined to the cutaneous wound sites, without histopathological evidence of invasive aspergillosis in the brain or in relation to the right ACA and MCA infarcts. These infarcts, however, may still represent vascular complications of angioinvasive *Aspergillus* infection, as embolization, vessel wall invasion, and thrombosis leading to cerebral infarction can occur even when fungal elements are not present in the examined tissue [1,4].

## 3 Discussion

Case reports describing *A. flavus* infection in neonates exist in the literature [[5], [6], [7],^10^]. Many of these reports highlight similar risk factors and frequently implicate nosocomial acquisition, particularly in preterm infants requiring intensive supportive care. In our case, the absence of fungal elements in the mother's placenta and the complete lack of disease in the monochorionic twin strongly suggest a postnatal, healthcare-associated source rather than congenital or peripartum transmission. Published reports also emphasize antifungal strategies but provide limited guidance on the nuances of cutaneous management in the setting of extensive fungal involvement.

Differentiating *Aspergillus* from *Mucorales* infections in neonates is difficult clinically and on frozen section. Definitive diagnosis requires culture. Dermatologic findings of neonatal aspergillosis include powder-like white rashes, yellow crusted, ulcerated lesions, or dark or target-like lesions [4]. *Mucorales* more often begin with erythema and induration progressing to necrosis with black eschar, often on covered skin or at trauma sites [4]. Histopathology distinguishes the two: *Aspergillus* is dichotomously branched with septate hyphae [8] whereas *Mucor* has broad non-septate or pauci-septate hyphae that branch at right angles from the parent hyphae [9].

Management hinges on early recognition and empiric antifungal therapy. Amphotericin B is the standard initial therapy, although voriconazole or echinocandins may be preferred once Aspergillus is confirmed [4,9,10]. Fluconazole lacks activity against *Mucor*, *Aspergillus* or *Candida krusei* although these infections are extremely rare in the neonate [4,9]. Combination therapy may be considered when diagnosis by culture has not been confirmed, though in vivo data suggests antagonism between the mechanism of action of amphotericin B and azoles, making amphotericin plus an echinocandin a more rational empiric regimen [10].

Biopsy should be pursued in target areas before full necrosis develops as this is critical for fungal detection and culture. Surgical debridement ultimately depends on progression of the disease and antifungals coverage and should focus on preserving function and preventing long-term disability [10].

An additional challenge is the increasing recognition of polyene resistance in *A.flavus* species as seen in our isolate with an amphotericin B minimum inhibitory concentration (MIC) of 4mcg/mL. Formal clinical breakpoints do not exist for this organism, although wild-type MIC distributions typically fall within the 0.5–2 mcg/mL range for amphotericin B, and values of ≥ 2–4 mcg/mL exceed the expected epidemiologic cutoff value [11]. In one Austrian cohort, in vitro susceptibility testing demonstrated approximately two-thirds of *A.flavus* isolates had elevated amphotericin B MICs, findings that correlated with clinical treatment failure [12]. Although limited by a small sample size, this study provided early evidence that polyene resistance is more common in *A.flavus* than *A.fumigatus*.

In addition to systemic antifungal therapy, local wound care remains a key supportive measure but comes with unique challenges due to skin immaturity and systemic safety concerns in preterm neonates. While topical amphotericin B has been described in case reports of invasive cutaneous mold, its use is not standardized in preterm infants, and in our setting was deemed neither feasible nor appropriate given lack of neonatal safety data. Silver sulfadiazine which is widely used in burn care has both antibacterial and antifungal activity (including activity against Candida and some filamentous fungi), was considered. However, its use in premature infants and those younger than 2 months is contraindicated due to systemic absorption and sulfonamide-associated risk of kernicterus, making it unsafe in this population [13,14]. Similarly, iodine-based antiseptics are avoided in preterm neonates because of transcutaneous absorption leading to transient hypothyroidism [15]. In practice, supportive measures like gentle cleansing, non-adherent dressings and meticulous skin protection remain the safest adjuncts to systemic antifungal therapy in these cases.

This case highlights the high mortality of *Aspergillus flavus* in extremely preterm infants despite early recognition, amphotericin B initiation and surgical debridement. It underscores the importance of multidisciplinary care for rapid biopsy, culture confirmation, and surgical debridement, as well as consideration of alternative antifungal regimens while also demonstrating the limitations of available therapies in this vulnerable population.

## CRediT authorship contribution statement

**Neema Pithia:** Writing – review & editing, Writing – original draft, Visualization, Investigation, Formal analysis, Conceptualization. **Margie Morgan:** Writing – review & editing, Visualization, Formal analysis. **Thea Tagliaferro:** Writing – review & editing, Visualization, Methodology, Investigation. **Priya R. Soni:** Writing – review & editing, Writing – original draft, Visualization, Supervision, Investigation, Formal analysis, Conceptualization.

## Ethical Form

Please note that this journal requires full disclosure of all sources of funding and potential conflicts of interest. The journal also requires a declaration that the author(s) have obtained written and signed consent to publish the case report report/case series from the patient(s) or legal guardian(s).

The statements on funding, conflict of interest and consent need to be submitted via our Ethical Form that can be downloaded from the submission site www.ees.elsevier.com/mmcr. **Please note that your manuscript will not be considered for publication until the signed Ethical Form has been received.**

## Conflict of interest

The authors declare that they have no known competing financial interests or personal relationships that could have appeared to influence the work reported in this paper.
